# Metabolic syndrome worsens sarcopenia and reduces nutritional therapy benefits in advanced gastric cancer

**DOI:** 10.3389/fnut.2025.1615376

**Published:** 2025-10-15

**Authors:** Lu Xu, Xinjie Zhang, Yuxin Feng, Vincent Kam Wai Wong, Wang Yao, Ying Feng

**Affiliations:** ^1^Department of Oncology, The Affiliated Suzhou Hospital of Nanjing Medical University, Suzhou Municipal Hospital, Suzhou, China; ^2^Dr. Neher’s Biophysics Laboratory for Innovative Drug Discovery, State Key Laboratory of Quality Research in Chinese Medicine, Faculty of Chinese Medicine, Macau University of Science and Technology, Macau, Macau SAR, China; ^3^Zhejiang Cancer Hospital, Hangzhou Institute of Medicine (HIM), Chinese Academy of Sciences, Hangzhou, China; ^4^Department of Cancer Center, The Fifth Affiliated Hospital of Wenzhou Medical University, Lishui, China

**Keywords:** metabolic syndrome, sarcopenia, Mendelian randomization, gastric cancer, nutritional therapy

## Abstract

**Background:**

Emerging evidence suggests metabolic syndrome (MetS) exacerbates sarcopenia progression and compromises nutritional interventions, yet its dual role as both etiological driver and therapeutic effect modifier remains uncharacterized. This study investigated MetS-related sarcopenia pathophysiology and assessed its impact on nutritional therapy efficacy in advanced gastric cancer.

**Patients and methods:**

We conducted a dual-phase investigation combining Mendelian randomization (MR) analysis of European-ancestry GWAS data (*n* = 654,783) with retrospective evaluation of 65 sarcopenic gastric cancer patients receiving chemotherapy and enteral nutrition. MR evaluated causal relationships between individual components of MetS and sarcopenia phenotypes, while clinical analyses compared outcomes by MetS status (IDF/AHA criteria).

**Results:**

MR analysis of MetS components identified paradoxical causal effects: waist circumference increased appendicular lean mass (OR = 1.480, *p* < 0.001) but impaired walking speed (OR = 0.864, *p* < 0.001). In the clinical cohort, MetS patients exhibited accelerated nutritional decline with 2.6-fold greater weight loss (−1.70 vs. − 0.66 kg, *p* = 0.01), attenuated muscle preservation (48.1% vs. 73.7% SMI improvement, *p* = 0.066), and reduced median PFS (75.0 vs. 84.5 days, *p* = 0.061). Protein trajectories revealed MetS-specific catabolic patterns, particularly transferrin depletion (*Δ* = -0.26 vs. − 0.05 g/L, *p* = 0.0004).

**Conclusion:**

The integration of genetic and clinical findings shows that MetS components causally contribute to sarcopenia pathogenesis, and that the composite MetS phenotype confers nutritional therapy resistance. This establishes MetS’s dual role as a driver of disease and a modifier of treatment efficacy.

## 1 Introduction

Sarcopenia, broadly characterized by the progressive loss of skeletal muscle mass and functional deterioration, is a prevalent comorbidity in cancer patients, affecting 30–60% of individuals across tumor types (1). It is strongly associated with increased chemotherapy toxicity, reduced treatment tolerance, and poorer survival outcomes (2, 3). While malnutrition has long been recognized as a key driver of sarcopenia, emerging evidence highlights metabolic dysregulation—particularly MetS, a cluster of conditions including central obesity, hypertension, and dyslipidemia—as an independent risk factor (4–6). According to the Asian-specific criteria established by the International Diabetes Federation (IDF) and the Asian Pacific Society of Cardiology (APSC), metabolic syndrome is defined by central obesity with ethnicity-adjusted waist circumference thresholds (≥90 cm for Asian men or ≥80 cm for Asian women), plus at least two of the following: elevated triglycerides, reduced HDL cholesterol, hypertension, or impaired fasting glucose (7, 8). These metabolic abnormalities share pathophysiological pathways with sarcopenia, such as chronic inflammation, mitochondrial dysfunction, and insulin resistance, creating a vicious cycle that exacerbates muscle catabolism (9).

Despite advances in understanding these interactions, critical gaps persist. While observational studies consistently associate MetS components with muscle loss (e.g., waist circumference, hypertension) and muscle loss (10, 11), the causal nature of this relationship remains uncertain due to inherent limitations of traditional epidemiological approaches (12). Furthermore, although enteral nutrition (EN) is widely recommended for cancer-related sarcopenia (13), its efficacy in patients with concurrent metabolic dysfunction requires clarification - particularly in gastric cancer where sarcopenia prevalence reaches 50% and significantly impacts the 350,000 annual new cases in China. While EN benefits early postoperative patients (14), its role in advanced disease, especially regarding potential mitigation of MetS-exacerbated muscle catabolism through inflammatory and insulin resistance pathways, remains poorly characterized.

To address these gaps, we employed a dual-method approach integrating causal inference with clinical validation. Using MR with genetic variants as instrumental variables, we established causal effects of MetS components on muscle function. Complementing these findings, our retrospective cohort study of advanced gastric cancer patients with sarcopenia evaluated EN efficacy stratified by MetS status. This integrated investigation not only clarifies the causal role of metabolic dysregulation in sarcopenia pathogenesis but also provides clinically actionable insights into how metabolic status modifies nutritional intervention outcomes, informing more personalized management strategies for cancer-associated sarcopenia.

## 2 Materials and methods

### 2.1 Study design and population

**Figure 1:**
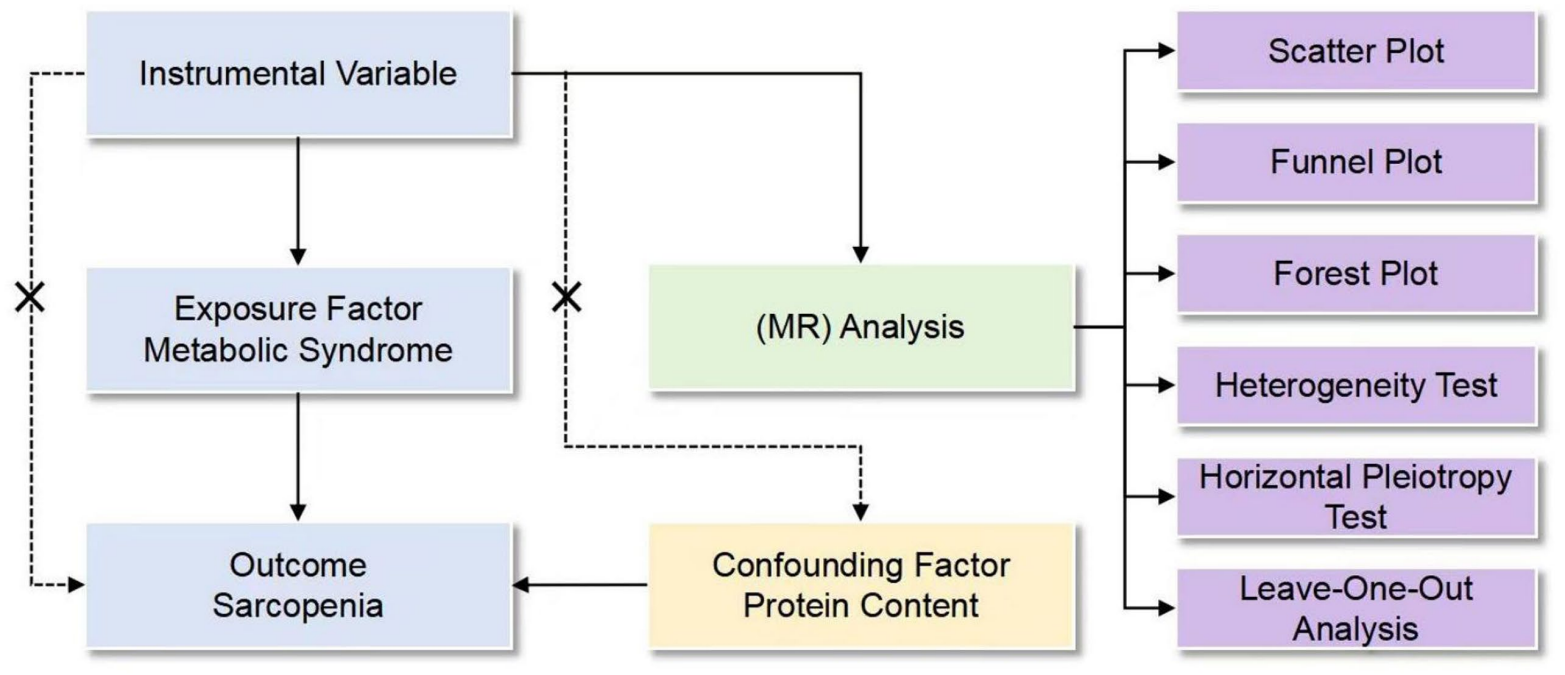
Design of MR analysis of the causal link between metabolic syndrome and sarcopenia.

This hybrid investigation employed MR analysis complemented by retrospective clinical validation to elucidate the MetS-sarcopenia relationship. The MR framework utilized genetic variants as instrumental variables (IVs), adhering to three core assumptions: IV-exposure association (correlation), IV-confounder independence (independence), and exclusion restriction (no direct IV-outcome effects) (15). Genetic instruments derived from European-ancestry GWAS datasets (CTGLAB/IEU OpenGWAS for MetS components; UK Biobank for sarcopenia phenotypes) ensured no sample overlap, with analytical framework detailed in Figure 1.

The clinical cohort comprised 65 stage IV gastric adenocarcinoma patients (AJCC 8th) with confirmed sarcopenia treated at Zhejiang Cancer Hospital (2019–2023), all receiving first-line therapy (chemotherapy/immunotherapy/targeted agents) combined with a standardized enteral nutrition protocol using Renon^®^. MetS diagnosis required ≥3 criteria: waist circumference ≥90/80 cm (M/F), fasting glucose ≥100 mg/dL/antidiabetic treatment, triglycerides ≥150 mg/dL/lipid therapy, HDL-C < 40/50 mg/dL (M/F), or BP ≥ 130/85 mmHg. The protocol (IRB-2024-710) received ethical approval with retrospective consent waiver.

### 2.2 Mendelian randomization analysis

#### 2.2.1 Data sources description

Genome-wide association study (GWAS) data were obtained from publicly available repositories. Exposure data encompassing genetic variants associated with MetS and its components were sourced from the CTGLAB and IEU OpenGWAS databases, comprising individuals of European ancestry. Outcome data for sarcopenia-related phenotypes, including hand grip strength (bilateral), appendicular lean mass, and walking speed, were derived from the UK Biobank resource (European participants). This study design ensured no sample overlap between exposure and outcome datasets, thereby minimizing potential bias. Comprehensive details regarding dataset characteristics are provided in Supplementary Table 1.

#### 2.2.2 Instrumental variable selection

IVs were selected through a rigorous multi-step process. Single nucleotide polymorphisms (SNPs) demonstrating significant genome-wide associations (*p* < 5 × 10^−8^) with exposure traits were initially identified (16). Linkage disequilibrium was addressed using a stringent threshold (r^2^ < 0.001) within a 10,000 kb clumping window (17). Instrument strength was validated by calculating F-statistics, with variants exhibiting *F* < 10 excluded to mitigate weak instrument bias (18). Potential confounding was minimized by screening the GWAS Catalog database (*p* < 1 × 10^−5^) to remove SNPs associated with outcome-related traits. The Steiger directionality test was applied to confirm correct causal orientation (19), and palindromic SNPs were harmonized between exposure and outcome datasets. Final IV characteristics are detailed in Supplementary Table 2.

#### 2.2.3 Statistical analysis and data visualization

Causal inference was performed using five complementary MR methods: inverse-variance weighted (IVW, primary analysis), MR-Egger, weighted median, simple mode, and weighted mode approaches (20). Model selection (fixed- vs. random-effects) was guided by Cochran’s Q test for heterogeneity (*p* < 0.05 indicating random-effects). Sensitivity analyses included: (1) MR-PRESSO for outlier detection and correction (Pdistortion < 0.05 considered significant); (2) MR-Egger intercept testing for horizontal pleiotropy; and (3) leave-one-out analysis to evaluate individual SNP influence (21). Effect estimates were expressed as odds ratios (OR) with 95% confidence intervals (CI). All analyses were conducted using R (version 4.2.1) with TwoSampleMR and MR-PRESSO packages.

### 2.3 Clinical cohort methods

#### 2.3.1 Study population

Eligible participants were required to meet four core criteria: (1) histologically confirmed stage IV gastric adenocarcinoma (AJCC 8th edition) with available pathology reports from diagnostic biopsies; (2) objectively diagnosed sarcopenia defined by L3-CT skeletal muscle index thresholds (≤40.8 cm^2^/m^2^ for males, ≤34.9 cm^2^/m^2^ for females) measured on baseline CT scans (22); (3) availability of ≥2 contrast-enhanced abdominal CT examinations performed at standardized 3-month intervals (±2 weeks) to ensure longitudinal muscle mass assessment; and (4) complete baseline metabolic syndrome profiling including centrally measured waist circumference, fasting glucose, lipid panel (triglycerides, HDL-C), and triplicate blood pressure recordings. All patients had received prior first-line systemic therapy (chemotherapy, immunotherapy, or targeted agents) by NCCN guidelines. Exclusion criteria addressed confounding through strict protocols: patients under 18 years, enteral nutrition interruption >7 consecutive days (medication administration records verified), recent use (≤6 months) of glucose-modifying agents (GLP-1 agonists, insulin sensitizers), decompensated hepatic/renal dysfunction (Child-Pugh C, eGFR <30 mL/min/1.73m^2^), active infections requiring antimicrobials, untreated endocrine disorders (TSH < 0.1 or >10 mIU/L), concurrent malignancies, or incomplete data (missing CT scans, metabolic parameters, or progression-free survival records).

#### 2.3.2 Nutritional intervention protocol

Enteral nutrition support was standardized for all participants using a commercially available, high-protein, peptide-based formula (Renon, Hangzhou Renon Pharmaceutical Co., Ltd., China). Administration was via nasoenteral tube, gastrostomy, or oral intake, with a target daily energy intake of 25–30 kcal/kg and protein intake of 1.5–2.0 g/kg (based on ideal body weight). The intervention began within 48 h of the first cycle of first-line systemic therapy and was continued throughout the treatment period or until nutritional status improved (defined as PG-SGA score ≤ 3). Adherence was monitored using electronic medical records and nursing charts. For most patients, the intervention lasted 3–4 months, aligning with imaging intervals and allowing sufficient time to evaluate physiological effects.

#### 2.3.3 Data collection and outcomes

Data acquisition followed a standardized protocol executed by two independent researchers blinded to clinical outcomes, involving systematic extraction from electronic medical records and PACS imaging archives. Baseline demographic parameters (age, sex, body mass index, waist circumference) and tumor characteristics (histological subtype, differentiation grade, TNM stage, metastatic patterns) were meticulously recorded. Metabolic syndrome profiling incorporated centrally measured waist circumference (midpoint between iliac crest and rib cage), fasting biochemical assays (glucose, triglycerides, HDL-C via enzymatic colorimetric methods), and triplicate blood pressure readings (seated position, Omron HEM-7320 sphygmomanometer) averaged for analysis. Serial nutritional assessments included serum total protein (biuret method), albumin (bromocresol green), prealbumin (immunoturbidimetry), and transferrin (nephelometry) measured on Roche Cobas^®^ platforms.

Muscle mass quantification utilized SliceOmatic^®^ v5.0 (TomoVision) under rigorous quality control: axial L3-level CT images were analyzed with standardized window settings (width 400 HU, level 40 HU), muscle attenuation thresholds (−29 to +150 HU), and semi-automated segmentation validated by dual radiologists (interclass correlation coefficient >0.90) (23). Primary endpoints encompassed body weight trajectories (kg), skeletal muscle index delta (ΔSMI = post-intervention - baseline), and longitudinal nutritional parameter trends. Secondary outcomes evaluated progression-free survival (PFS), defined as the time from treatment initiation to radiologically confirmed progression (RECIST 1.1) or death. PFS assessments were adjudicated by treating physicians based on integrated radiological and clinical evaluations.

#### 2.3.4 Statistical analysis

Continuous variables were analyzed using parametric or nonparametric methods based on distributional assumptions. Longitudinal outcomes were modeled via linear mixed-effects regressions with time, metabolic syndrome (MetS) status, and their interaction as fixed effects. These models were adjusted for pre-specified potential confounders identified *a priori* based on clinical relevance, including age, sex, and baseline body mass index (BMI). Participant-specific intercepts were included as random effects, with Kenward-Roger degrees of freedom estimation. Categorical variables were assessed using χ^2^/Fisher’s exact tests (effect sizes: Cramér’s V). Survival endpoints (e.g., progression-free survival) were analyzed by Kaplan–Meier/log-rank tests and Cox proportional hazards models. The Benjamini-Hochberg procedure controlled the false discovery rate (FDR ≤ 5%). Statistical analyses were conducted in SPSS 26.0 and GraphPad Prism 9.0.

## 3 Results

### 3.1 Mendelian randomization analysis of metabolic syndrome components

The MR analysis results for exposures and their effects on left-hand grip strength, right-hand grip strength, and appendicular lean mass are shown. A *p*-value < 0.05 indicates a significant causal relationship. IVW analysis showed that a significant causal relationship was observed for waist circumference, which was positively associated with left-hand grip strength (OR = 1.076 (1.018 to 1.136) *p* < 0.05). Similarly, waist circumference showed a significant positive correlation with right-hand grip strength (OR = 1.068 (1.014 to 1.126), *p* < 0.05). In the analysis of appendicular lean mass, waist circumference showed a significant positive correlation (OR = 1.480 (1.283 to 1.707), *p* < 0.05). However, despite demonstrating a positive correlation, metabolic syndrome exhibited horizontal pleiotropy (pleio_*P* < 0.05) and was consequently excluded from subsequent analysis. The outcomes of these analyses are shown in Table 1.

**Table 1:** Mendelian randomization results for the exposure and outcome (left-hand grip strength, right-hand grip strength, and appendicular lean mass).

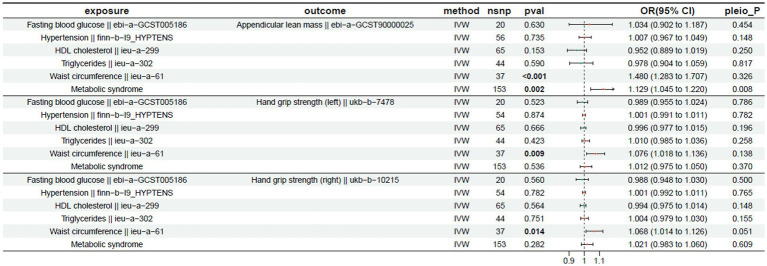

Exposure: The risk factor or exposure of interest; Outcome: The outcome or result being studied; Method: The analytical approach or method used; nsnp: The number of instrumental variables used in the analysis; pval: *p*-value, indicating statistical significance; OR: Odds ratio, with the 95% confidence interval for the odds ratio in parentheses; pleio_P: *p*-value for the pleiotropy test, assessing horizontal pleiotropic effects.

The MR results for the exposure factors and usual walking speed are presented in Table 2. A *p*-value < 0.05 indicates a significant causal relationship. Hypertension showed a significant negative correlation with usual walking speed (OR = 0.985 (0.976 to 0.993), *p* < 0.05). Waist circumference was also exhibited a significant negative correlation with walking speed (OR = 0.864 (0.832 to 0.898), *p* < 0.05). Furthermore, metabolic syndrome exhibited a significant negative correlation with walking speed (OR = 0.809 (0.790 to 0.829), *p* < 0.05).

**Table 2:** Mendelian randomization results for the exposure and outcome (usual walking pace).

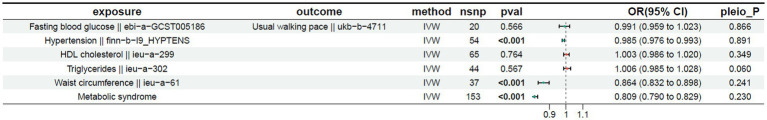

### 3.2 Impact on physical function

In the analysis of sarcopenia-related outcomes, MR analysis revealed that individual components of MetS were significantly negatively associated with walking speed. These findings indicate that genetic predisposition to higher waist circumference and hypertension may lead to a decrease in walking speed, thereby identifying them as potential causal risk factors for impaired physical function.

For the outcomes of appendicular lean mass and grip strength, a divergent causal pattern was observed. Waist circumference showed a significant positive causal relationship with both left-hand grip strength, right-hand grip strength, and appendicular lean mass. The coexistence of these opposing causal effects—whereby genetic predisposition to higher waist circumference increases muscle mass and strength but decreases walking speed—highlights a critical dissociation between muscle quantity and physical function.

**Figure 2:**
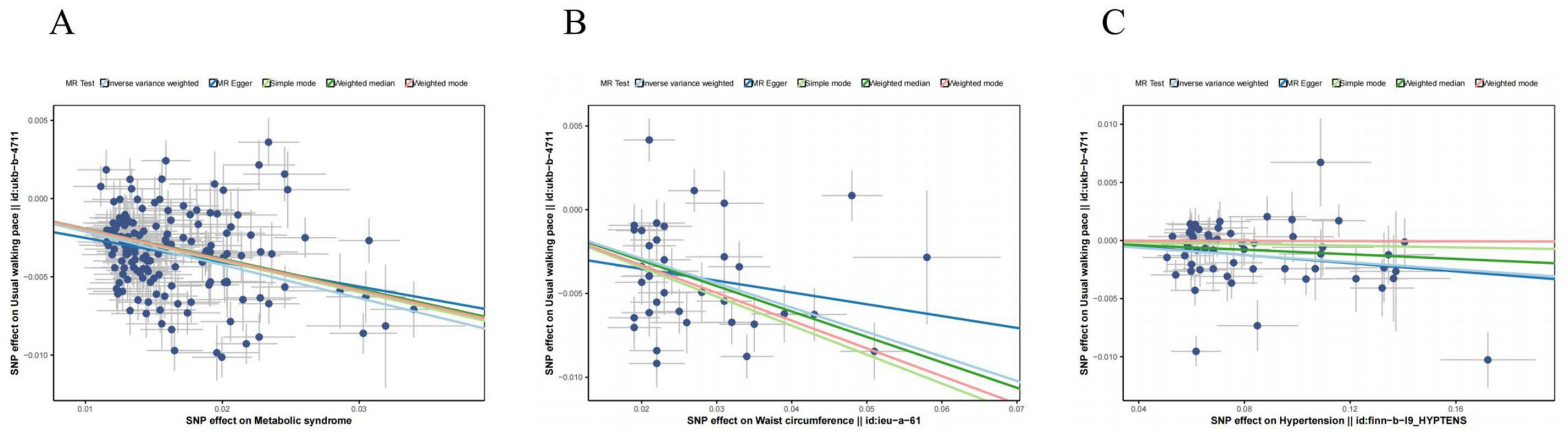
Scatter plot of the causality of MetS on sarcopenia. **(A)** Scatter plot of the causality of metabolic syndrome on usual walking pace. **(B)** Scatter plot of the causality of waist circumference on usual walking pace. **(C)** Scatter plot of the causality of hypertension on usual walking pace.

**Figure 3:**
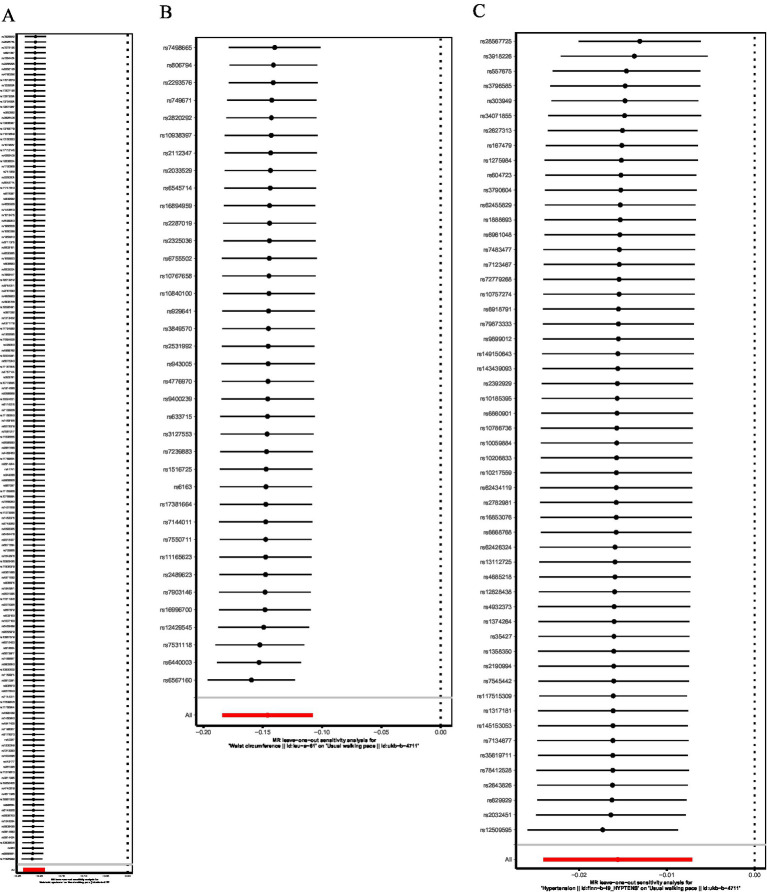
Leave-one-out of the effect of MetS on sarcopenia. **(A)** Leave-one-out of the effect of metabolic syndrome on usual walking pace. **(B)** Leave-one-out of the effect of waist circumference on usual walking pace. **(C)** Leave-one-out of the effect of hypertension on usual walking pace.

This apparent paradox underscores the complexity of the relationship between adiposity and musculoskeletal health. The significant associations for walking speed are visually depicted in scatter plots derived from five MR methods (Figure 2), while leave-one-out sensitivity analyses confirmed the robustness of all causal estimates (Figure 3).

### 3.3 Clinical cohort characteristics

The study included 65 patients with metastatic gastric cancer (TNM stage IV, M1) and sarcopenia, stratified by MetS status (MetS: *n* = 27; non-MetS: *n* = 38) according to IDF/AHA criteria (Table 3). Baseline characteristics showed no significant differences in age (60.1 ± 10.3 vs. 62.2 ± 7.8 years, *p* = 0.257), sex distribution (50.0% vs. 59.3% female, *p* = 0.627), or tumor differentiation (34.2% vs. 51.9% G1, *p* = 0.362). MetS patients had significantly higher waist circumference (88.2 ± 5.2 vs. 85.8 ± 4.8 cm, *p* = 0.048), fasting glucose (111.8 ± 18.5 vs. 101.2 ± 20.8 mg/dL, *p* = 0.006), systolic blood pressure (135.6 ± 10.0 vs. 129.6 ± 8.4 mmHg, *p* = 0.006), and lower HDL-C (47.4 ± 9.3 vs. 52.9 ± 10.0 mg/dL, *p* = 0.036), with borderline elevated triglycerides (154.3 ± 20.1 vs. 141.6 ± 27.8 mg/dL, *p* = 0.059). All patients had metastatic disease (T3:64.6%, T4:35.4%; N2:6.2%, N3:21.5%), with comparable T-stage (65.8% vs. 63.0% T3, *p* > 0.999) and N-stage (26.3% vs. 14.8% N3, *p* = 0.528) between groups. Histopathology showed predominantly adenocarcinoma (90.8%), with signet ring cell carcinoma equally distributed (9.2%, *p* > 0.999).

**Table 3:** Baseline characteristics by MetS status.

Variables	Total (*N* = 65)	Non-MetS group (*N* = 38)	MetS group (*N* = 27)	*p*-value
Age	61.0 ± 9.3	60.1 ± 10.3	62.2 ± 7.8	0.257
Sex				0.627
Female	35 (53.8%)	19 (50.0%)	16 (59.3%)	
Male	30 (46.2%)	19 (50.0%)	11 (40.7%)	
Pathology				>0.999
AD	59 (90.8%)	34 (89.5%)	25 (92.6%)	
SRCC	6 (9.2%)	4 (10.5%)	2 (7.4%)	
Differ				0.362
G1	27 (41.5%)	13 (34.2%)	14 (51.9%)	
G2	15 (23.1%)	10 (26.3%)	5 (18.5%)	
G3	23 (35.4%)	15 (39.5%)	8 (29.6%)	
T				>0.999
III	42 (64.6%)	25 (65.8%)	17 (63.0%)	
IV	23 (35.4%)	13 (34.2%)	10 (37.0%)	
N				0.528
N2	4 (6.2%)	2 (5.3%)	2 (7.4%)	
N3	14 (21.5%)	10 (26.3%)	4 (14.8%)	
Nx	47 (72.3%)	26 (68.4%)	21 (77.8%)	
MetS status				
Waist	86.8 ± 5.1	85.8 ± 4.8	88.2 ± 5.2	0.048*
FBG	105.6 ± 20.4	101.2 ± 20.8	111.8 ± 18.5	0.006**
TG	146.9 ± 25.5	141.6 ± 27.8	154.3 ± 20.1	0.059
HDL-C	50.6 ± 10.0	52.9 ± 10.0	47.4 ± 9.3	0.036*
SBP	132.1 ± 9.5	129.6 ± 8.4	135.6 ± 10.0	0.006**

Mean ± SD; n (%); Wilcoxon rank sum test; Pearson’s Chi-squared test. *p < 0.05, **p < 0.01.

### 3.4 Nutritional intervention outcomes

**Figure 4:**
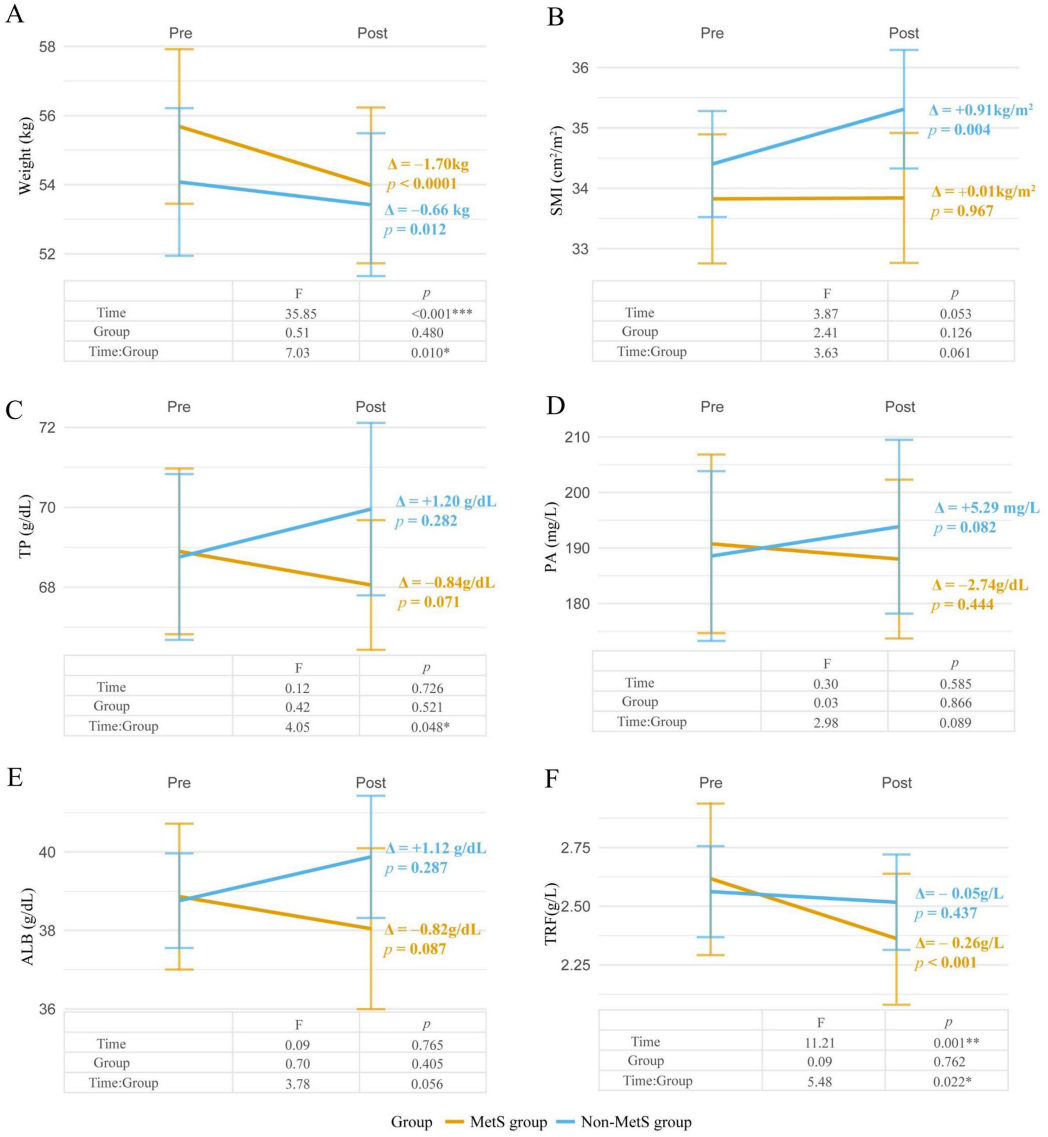
Time-dependent changes and group differences in nutrition-related parameters between MetS and non-MetS groups. **(A)** Weight: interaction effect of time and group. **(B)** Skeletal muscle index (SMI): interaction effect of time and group. **(C)** Total Protein (TP): interaction effect of time and group. **(D)** Albumin (ALB): interaction effect of time and group. **(E)** Prealbumin (PA): interaction effect of time and group. **(F)** Transferrin (TRF): Interaction Effect of Time and Group. Longitudinal changes in nutritionrelated parameters are compared between MetS (yellow) and non-MetS (blue) groups. Statistical significance was assessed via repeated-measures ANOVA, reporting *F*-values (*F*-statistic for interaction effects) and p-values for Time, Group, and Time × Group interactions. *p < 0.05, **p < 0.01, ***p < 0.001, error bars represent standard deviation.

The baseline clinical characteristics and distribution of post-treatment parameters across study groups are detailed in Supplementary Table 3. Therapeutic efficacy analysis revealed significant MetS-dependent divergence in body composition and nutritional biomarkers (Figure 4; Table 4). Linear mixed models demonstrated universal weight reduction across all patients (time effect: *F* = 28.4, *p* < 0.001), with MetS patients exhibiting 2.6-fold greater weight loss (−1.70 kg vs. − 0.66 kg; MetS×time interaction *F* = 7.03, *p* = 0.01). Skeletal muscle responses showed differential preservation patterns: non-MetS patients gained 0.91 cm^2^/m^2^ in SMI (*p* = 0.004) versus MetS stagnation (*Δ* = +0.02 cm^2^/m^2^, *p* = 0.967), with borderline time-by-MetS interaction (*F* = 3.63, *p* = 0.061). Categorical analysis confirmed disproportionate deterioration in MetS group (85% weight loss vs. 42% non-MetS; χ^2^ = 8.39, *p* = 0.015), paralleled by divergent SMI trajectories (73.8% non-MetS improvement vs. 48.1% MetS; χ^2^ = 3.39, *p* = 0.066).

**Table 4:** Weight and skeletal muscle index (SMI) trajectories by metabolic syndrome status.

Variables	Non-MetS group (*N* = 38)	MetS group (*N* = 27)	*p*-value
Weight change categories			0.015**
Increase (>2%)	4 (10.5%)	1 (3.7%)	
Stable (±2%)	26 (68.4%)	11 (40.7%)	
Decrease (>2%)	8 (21.1%)	15 (55.6%)	
SMI trajectory groups			0.066
Up	28 (73.7%)	13 (48.1%)	
Down	10 (26.3%)	14 (51.9%)	

n (%); Pearson’s Chi-squared test. *p < 0.05, **p < 0.01.

Protein trajectories exhibited distinct metabolic modulation (Figures 4C–F): While non-MetS patients demonstrated marginal increases in total protein (*Δ* = +1.20 g/L, *p* = 0.071) and prealbumin (Δ = +5.29 mg/L, *p* = 0.082), MetS counterparts showed paradoxical declines (Δ = -0.84 g/L, *p* = 0.282; Δ = -2.74 mg/L, *p* = 0.443), with significant interaction effects for total protein (*F* = 4.05, *p* = 0.049) and borderline prealbumin interaction (*F* = 2.98, *p* = 0.089). Albumin trajectories mirrored this pattern (non-MetS Δ = +1.12 g/dL, *p* = 0.087 vs. MetS Δ = -0.82 g/dL, *p* = 0.287; interaction *F* = 3.78, *p* = 0.056). Strikingly, transferrin displayed MetS-specific depletion (Δ = -0.26 g/L, *p* = 0.0004) contrasting with non-MetS stability (Δ = -0.05 g/L, *p* = 0.437), demonstrating the strongest metabolic interaction (*F* = 5.48, *p* = 0.022).

### 3.5 Progression-free survival (PFS)

**Figure 5:**
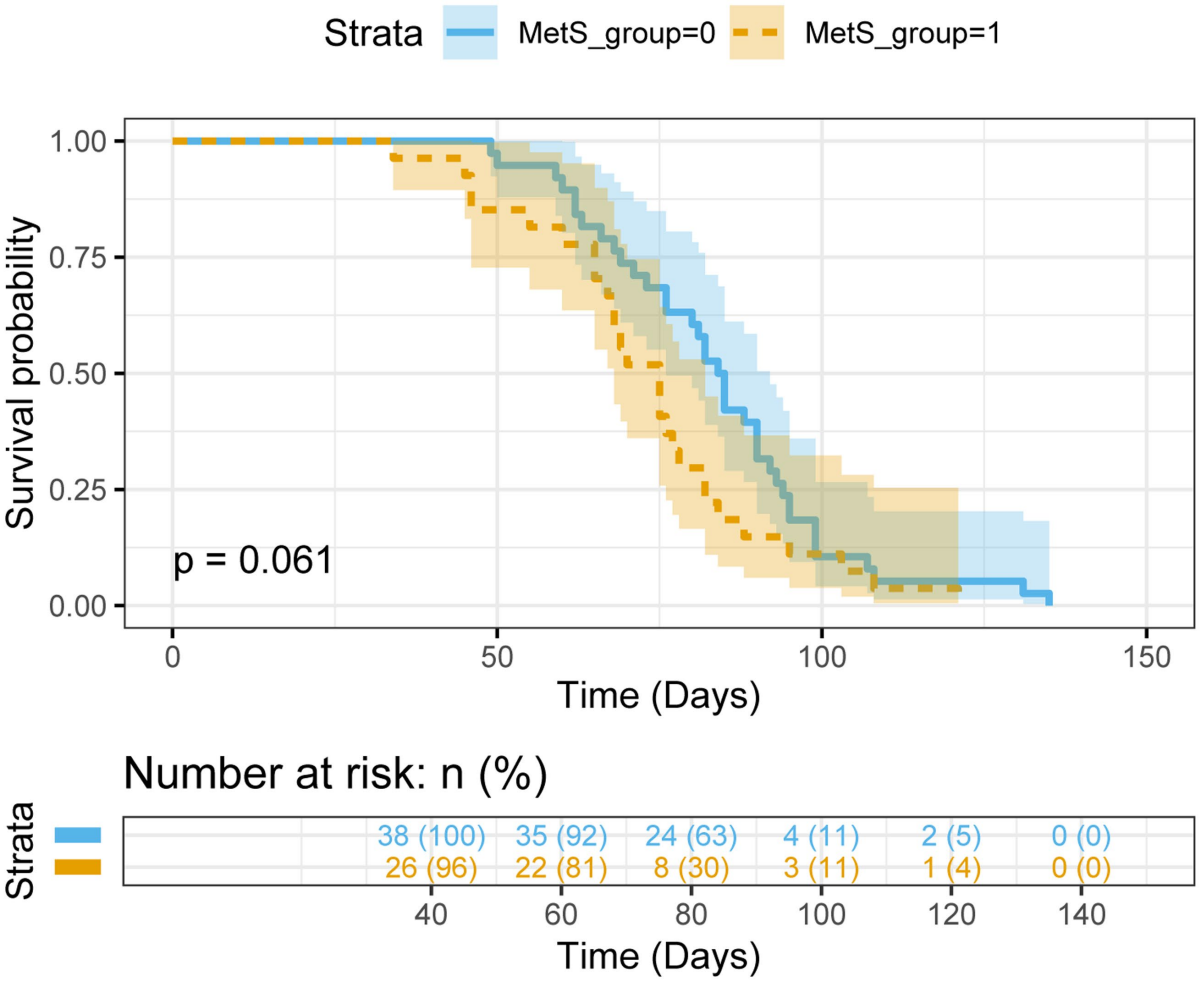
PFS in gastric cancer (GC) patients stratified by metabolic syndrome (MetS) status.

All enrolled patients experienced disease progression or death (PFS event rate 100%). The non-MetS group exhibited a median PFS of 84.5 days (95% CI: 76–92), compared to 75.0 days (95% CI: 68–82) in MetS patients, representing a clinically relevant 9.5-day survival disadvantage for MetS patients. Kaplan–Meier analysis (Figure 5) demonstrated progressive separation of survival curves, with log-rank testing non-significant trend (χ^2^ = 3.52, *p* = 0.061). This 12.7% reduction in median PFS suggests accelerated disease progression in MetS patients despite standardized chemotherapy and nutritional intervention.

## 4 Discussion

This integrated investigation employing MR and clinical cohort analyses provides novel insights into the dual role of MetS as both an etiological driver and therapeutic effect modifier in sarcopenia pathogenesis. Our MR analysis, focused on individual components of MetS, revealed a defining paradox: genetic predisposition to higher waist circumference exerted dual causal effects, increasing appendicular lean mass and grip strength yet simultaneously decreasing walking speed. A similar detrimental causal effect was observed for hypertension on walking speed. Our clinical findings further demonstrated that MetS is associated with preserved muscle mass but attenuated therapeutic benefits. These observations challenge conventional sarcopenia paradigms and underscore the complex interplay between metabolic dysregulation and muscle biology. A key consideration is the difference in populations between the genetic (European-ancestry GWAS) and clinical (Chinese cohort) analyses. While this precludes direct generalization, the biological pathways implicated are fundamental across populations. Thus, the clinical findings demonstrate that the pathophysiological process, for which MR provides causal evidence at the component level, is operational and impactful as a syndromic entity in a distinct, high-risk patient setting.

The sarcopenic obesity paradox provides the key conceptual framework for reconciling these seemingly discordant results. The detrimental impact of MetS components on walking speed aligns with established mechanisms linking insulin resistance and chronic inflammation to muscle quality decline. Specifically, the robust association between hypertension and functional impairment (OR = 0.985) suggests vascular mechanisms may underpin sarcopenia pathogenesis, where endothelial dysfunction and capillary rarefaction compromise muscle perfusion—a hypothesis supported by recent demonstrations of impaired nitric oxide bioavailability in MetS-related myopathy (24). The paradoxical increase in appendicular lean mass (OR = 1.480), rather than being an outlier, is a hallmark of this paradox, where greater absolute muscle mass may not confer functional benefit due to often concomitant impairments in muscle quality. This finding likely reflects adipose-muscle crosstalk, where visceral fat-derived follistatin-like 1 (FSTL1) antagonizes myostatin activity to promote muscle hypertrophy, albeit at the cost of inducing insulin resistance (25). This metabolic trade-off creates a “pseudo-sarcopenic” phenotype where quantitative muscle mass metrics mask functional deficits, necessitating revised diagnostic criteria incorporating dynamometric assessments.

Clinical analyses incorporating fixed effects of time, metabolic syndrome status, and their interactions revealed significant therapeutic response heterogeneity across key parameters. Weight, total protein (TP), and transferrin (TRF) demonstrated robust MetS-dependent differential responses (*p*_interaction_<0.05), while skeletal muscle index (SMI), prealbumin (PA), and albumin (ALB) showed non-significant trends for interactions (0.05 < *p*_interaction_<0.10), collectively indicating MetS attenuates therapeutic efficacy across nutritional and musculoskeletal metrics. The diminished therapeutic efficacy in MetS patients was most pronounced in weight dynamics (−1.70 kg vs. −0.66 kg loss) and iron metabolism, evidenced by MetS-specific transferrin depletion (*Δ* = −0.26 vs. −0.05 g/L), likely mediated through chronic inflammation-driven hepcidin overexpression that restricts iron mobilization (26). Concurrent declines in hepatic synthetic markers—albumin (Δ = −0.82 g/dL) and prealbumin (Δ = −2.74 mg/L)—mirror patterns observed in NAFLD progression (27), suggesting MetS exacerbates subclinical liver dysfunction. These proteomic perturbations coalesce into a malnutrition-inflammation axis where sustained catabolic signaling, potentially via IL-6-mediated JAK/STAT activation (28), overrides nutritional anabolism, establishing MetS as both a biological filter and amplifier of therapeutic resistance.

Although our clinical cohort could not directly assess physical function, the observed MetS-associated resistance to nutritional therapy—manifested as attenuated muscle mass preservation and profound catabolism—likely represents the physiological counterpart to the impairment in walking speed identified by the MR analysis. Both findings converge to indicate that MetS predisposes to a more severe and treatment-refractory sarcopenia phenotype. This is further reflected in the 9.5-day PFS reduction in MetS patients (75.0 vs. 84.5 days), which extends the clinical implications beyond sarcopenia management to cancer therapeutics. We postulate that MetS-induced gut barrier dysfunction (29) may alter chemotherapeutic agent bioavailability, while elevated free fatty acids compete with albumin-bound chemotherapeutic agents (e.g., taxanes, irinotecan) for protein binding (30)—mechanisms requiring validation through pharmacokinetic studies. The differential toxicity profiles observed suggest metabolic status should inform risk stratification in treatment protocols.

**Figure 6:**
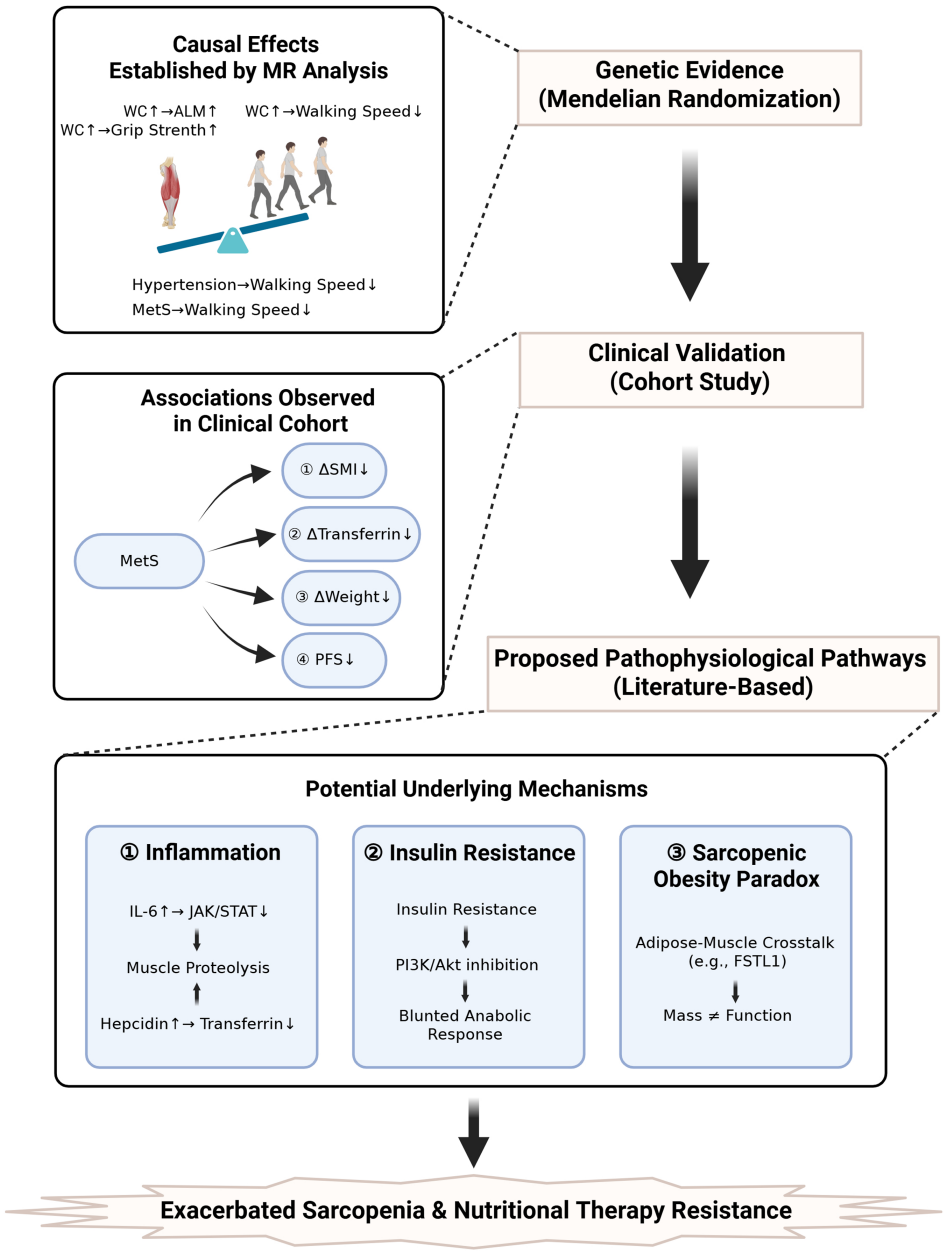
Integrated summary of causal and clinical findings with proposed mechanisms. ALM, appendicular lean mass; MetS, metabolic syndrome; PFS, progression-free survival; WC, waist circumference.

The integrative pathophysiological model presented in Figure 6 provides a framework for understanding how MetS creates a biological context that exacerbates sarcopenia and confers resistance to nutritional therapy, ultimately impacting cancer outcomes.

Several limitations warrant consideration. First, the European ancestry of MR data limits the generalizability of the genetic findings to diverse populations. Second, the clinical cohort’s modest sample size, particularly in the MetS subgroup (*n* = 27), reduces statistical power and increases the risk of Type II errors. As a result, the observed non-significant trends (e.g., in SMI and PFS]) should be interpreted as hypothesis-generating rather than conclusive findings. Third, the narrow patient population (advanced metastatic gastric cancer with sarcopenia) limits external validity to other cancer types or stages. Fourth, the retrospective design necessitated reliance on CT-based sarcopenia criteria, which precluded assessment of muscle function and direct validation of the MR-based functional outcomes.

Future investigation should prioritize multi-ethnic MR analyses to disentangle genetic vs. environmental MetS effects and incorporate objective functional assessments like gait speed into sarcopenia diagnostics. Furthermore, future studies should incorporate assessments of metabolic dysfunction-associated steatotic liver disease (MASLD), utilizing promising biomarkers such as plasma homocysteine and the atherogenic index of plasma to elucidate its shared mechanisms with sarcopenia (31, 32). Mechanistic studies exploring adipose-derived exosomes’ role in muscle metabolism could clarify the obesity paradox. Clinically, trials combining GLP-1 agonists with high-protein nutritional support may counter MetS-related anabolic resistance, leveraging their dual metabolic and anti-inflammatory properties.

In conclusion, this study repositions MetS as both a catalyst and amplifier of sarcopenia pathophysiology. The findings mandate a paradigm shift in sarcopenia management—from generic nutritional support to precision strategies addressing individual metabolic profiles. While enteral nutrition remains foundational, its efficacy appears contingent on metabolic health status, underscoring the imperative for combinatorial approaches targeting insulin signaling, inflammation, and mitochondrial function in high-risk MetS populations.

## Data Availability

The original contributions presented in the study are included in the article/Supplementary material, further inquiries can be directed to the corresponding authors.

## References

[1] Anjanappa M Corden M Green A Roberts D Hoskin P McWilliam A. Sarcopenia in cancer: risking more than muscle loss. Tech Innov Patient Support Radiat Oncol. (2020) 16:50–7. doi: 10.1016/j.tipsro.2020.10.001, PMID: 33385074 PMC7769854

[2] Bozzetti F. Forcing the vicious circle: sarcopenia increases toxicity, decreases response to chemotherapy and worsens with chemotherapy. Ann Oncol. (2017) 28:2107–18. doi: 10.1093/annonc/mdx271, PMID: 28911059

[3] Ryan AM Prado CM Sullivan ES Power DG Daly LE. Effects of weight loss and sarcopenia on response to chemotherapy, quality of life, and survival. Nutrition. (2019) 68:110539. doi: 10.1016/j.nut.2019.06.020 31522087

[4] Lee DY Shin S. Sarcopenia is associated with metabolic syndrome in Korean adults aged over 50 years: a cross-sectional study. Int J Environ Res Public Health. (2022) 19:1330. doi: 10.3390/ijerph19031330, PMID: 35162353 PMC8835141

[5] Cheng Q Wu C Guo L Hu J. Editorial: the relationship between sarcopenia and metabolic diseases: its formation mechanism and intervention means. Front Endocrinol. (2022) 13:972238. doi: 10.3389/fendo.2022.972238, PMID: 36093114 PMC9453858

[6] Agarwal K Saikia P Podder I. Metabolic syndrome and dyslipidemia in xanthelasma palpebrarum and associated risk-2 factors-a case-control study. J Cosmet Dermatol. (2022) 21:7018–24. doi: 10.1111/jocd.15353, PMID: 36057448

[7] Alberti KG Zimmet P Shaw J. Metabolic syndrome--a new world-wide definition. A consensus statement from the international diabetes federation. Diabet Med. (2006) 23:469–80. doi: 10.1111/j.1464-5491.2006.01858.x, PMID: 16681555

[8] Alberti KG Eckel RH Grundy SM Zimmet PZ Cleeman JI Donato KA. Harmonizing the metabolic syndrome: a joint interim statement of the international diabetes federation task force on epidemiology and prevention; National Heart, Lung, and Blood Institute; American Heart Association; world heart federation; international atherosclerosis society; and International Association for the Study of obesity. Circulation. (2009) 120:1640–5. doi: 10.1161/CIRCULATIONAHA.109.192644, PMID: 19805654

[9] Kim YC Ki SW Kim H Kang S Kim H Go GW. Recent advances in nutraceuticals for the treatment of Sarcopenic obesity. Nutrients. (2023) 15:854. doi: 10.3390/nu15173854, PMID: 37686886 PMC10490319

[10] Zhang H Lin S Gao T Zhong F Cai J Sun Y. Association between sarcopenia and metabolic syndrome in middle-aged and older non-obese adults: a systematic review and Meta-analysis. Nutrients. (2018) 10:364. doi: 10.3390/nu10030364, PMID: 29547573 PMC5872782

[11] Jiang M Ren X Han L Zheng X. Associations between sarcopenic obesity and risk of cardiovascular disease: a population-based cohort study among middle-aged and older adults using the CHARLS. Clin Nutr. (2024) 43:796–802. doi: 10.1016/j.clnu.2024.02.002, PMID: 38350287

[12] Zheng J Baird D Borges MC Bowden J Hemani G Haycock P. Recent developments in Mendelian randomization studies. Curr Epidemiol Rep. (2017) 4:330–45. doi: 10.1007/s40471-017-0128-6, PMID: 29226067 PMC5711966

[13] Arends J Bachmann P Baracos V Barthelemy N Bertz H Bozzetti F. ESPEN guidelines on nutrition in cancer patients. Clin Nutr. (2017) 36:11–48. doi: 10.1016/j.clnu.2016.07.015, PMID: 27637832

[14] Triantafillidis JK Papakontantinou J Antonakis P Konstadoulakis MM Papalois AE. Enteral nutrition in operated-on gastric Cancer patients: an update. Nutrients. (2024) 16:639. doi: 10.3390/nu16111639, PMID: 38892572 PMC11174039

[15] Taylor K Wootton RE Yang Q Oddie S Wright J Yang TC. The effect of maternal BMI, smoking and alcohol on congenital heart diseases: a Mendelian randomisation study. BMC Med. (2023) 21:35. doi: 10.1186/s12916-023-02731-y, PMID: 36721200 PMC9890815

[16] Michaëlsson M Yuan S Melhus H Baron JA Byberg L Larsson SC. The impact and causal directions for the associations between diagnosis of ADHD, socioeconomic status, and intelligence by use of a bi-directional two-sample Mendelian randomization design. BMC Med. (2022) 20:106. doi: 10.1186/s12916-022-02314-3, PMID: 35399077 PMC8996513

[17] Yan T Zhu S Xie C Zhu M Weng F Wang C. Coronary artery disease and atrial fibrillation: a bidirectional Mendelian randomization study. J Cardiovasc Dev Dis. (2022) 9:69. doi: 10.3390/jcdd9030069, PMID: 35323617 PMC8949548

[18] Yun Z Nan M Li X Liu Z Xu J Du X. Processed meat, red meat, white meat, and digestive tract cancers: a two-sample Mendelian randomization study. Front Nutr. (2023) 10:1078963. doi: 10.3389/fnut.2023.1078963, PMID: 36860687 PMC9968810

[19] Xia K Wang Y Zhang L Tang L Zhang G Huang T. Dietary-derived essential nutrients and amyotrophic lateral sclerosis: a two-sample Mendelian randomization study. Nutrients. (2022) 14:20. doi: 10.3390/nu14050920, PMID: 35267896 PMC8912818

[20] Zou L Guo H Berzuini C. Bayesian mendelian randomization with study heterogeneity and data partitioning for large studies. BMC Med Res Methodol. (2022) 22:162. doi: 10.1186/s12874-022-01619-4, PMID: 35658839 PMC9164425

[21] Park KW Hwang YS Lee SH Jo S Chung SJ. The effect of blood lipids, type 2 diabetes, and body mass index on Parkinson's disease: a Korean Mendelian randomization study. J Mov Disord. (2023) 16:79–85. doi: 10.14802/jmd.22175, PMID: 36628424 PMC9978253

[22] Chen XY Li B Ma BW Zhang XZ Chen WZ Lu LS. Sarcopenia is an effective prognostic indicator of postoperative outcomes in laparoscopic-assisted gastrectomy. Eur J Surg Oncol. (2019) 45:1092–8. doi: 10.1016/j.ejso.2018.09.030, PMID: 30853168

[23] Zeng X Shi ZW Yu JJ Wang LF Luo YY Jin SM. Sarcopenia as a prognostic predictor of liver cirrhosis: a multicentre study in China. J Cachexia Sarcopenia Muscle. (2021) 12:1948–58. doi: 10.1002/jcsm.12797, PMID: 34520115 PMC8718091

[24] Muniyappa R Chen H Montagnani M Sherman A Quon MJ. Endothelial dysfunction due to selective insulin resistance in vascular endothelium: insights from mechanistic modeling. Am J Physiol Endocrinol Metab. (2020) 319:E629–e646. doi: 10.1152/ajpendo.00247.2020, PMID: 32776829 PMC7642854

[25] Xu X Zhang T Mokou M Li L Li P Song J. Follistatin-like 1 as a novel Adipomyokine related to insulin resistance and physical activity. J Clin Endocrinol Metab. (2020) 105:e4499–509. doi: 10.1210/clinem/dgaa629, PMID: 32894773

[26] Ganz T Nemeth E. Iron homeostasis in host defence and inflammation. Nat Rev Immunol. (2015) 15:500–10. doi: 10.1038/nri3863, PMID: 26160612 PMC4801113

[27] Ipsen DH Lykkesfeldt J Tveden-Nyborg P. Molecular mechanisms of hepatic lipid accumulation in non-alcoholic fatty liver disease. Cell Mol Life Sci. (2018) 75:3313–27. doi: 10.1007/s00018-018-2860-6, PMID: 29936596 PMC6105174

[28] Hu W Ru Z Zhou Y Xiao W Sun R Zhang S. Lung cancer-derived extracellular vesicles induced myotube atrophy and adipocyte lipolysis via the extracellular IL-6-mediated STAT3 pathway. Biochim Biophys Acta Mol Cell Biol Lipids. (2019) 1864:1091–102. doi: 10.1016/j.bbalip.2019.04.006, PMID: 31002945

[29] Yang S Hu T Liu H Lv YL Zhang W Li H. Akebia saponin D ameliorates metabolic syndrome (MetS) via remodeling gut microbiota and attenuating intestinal barrier injury. Biomed Pharmacother. (2021) 138:111441. doi: 10.1016/j.biopha.2021.111441, PMID: 33652261

[30] Venianakis T Primikyri A Opatz T Petry S Papamokos G Gerothanassis IP. NMR and docking calculations reveal novel atomistic selectivity of a synthetic high-affinity free fatty acid vs. free fatty acids in Sudlow's drug binding sites in human serum albumin. Molecules. (2023) 28:991. doi: 10.3390/molecules28247991, PMID: 38138481 PMC10745614

[31] De Matteis C Crudele L Di Buduo E Cantatore S Gadaleta RM Cariello M. Hyperhomocysteinemia is linked to MASLD. Eur J Intern Med. (2025) 131:49–57. doi: 10.1016/j.ejim.2024.10.014 39482164

[32] De Matteis C Novielli F Di Buduo E Arconzo M Gadaleta RM Cariello M. Atherogenic index of plasma identifies subjects with severe liver steatosis. Sci Rep. (2025) 15:9136. doi: 10.1038/s41598-025-93141-y, PMID: 40097487 PMC11914574

