# Supplementary material for: Metabolic syndrome worsens sarcopenia and reduces nutritional therapy benefits in advanced gastric cancer
Source: Front Nutr. 2025 Oct 15;12:1615376. doi: 10.3389/fnut.2025.1615376 (PMC12568025; doi:10.3389/fnut.2025.1615376)
Supplement: Supplementary file 1 [file Data_Sheet_1.ZIP › supplementary tables 2025.8.24/supplementary table 3.docx]

**supplementary table 3. Baseline and Post-Intervention Characteristics by Metabolic Syndrome Status**

| **Variables** | **Non-MetS Group**  **(N=38)** | **MetS Group**  **(N=27)** | ***p*-value** |
| --- | --- | --- | --- |
| Weight_1 | 54.1 ± 6.5 | 55.7 ± 5.7 | 0.150 |
| Weight_2 | 53.4 ± 6.3 | 54.0 ± 5.7 | 0.295 |
| BMI_1 | 20.5 ± 1.8 | 21.4 ± 1.5 | 0.022* |
| BMI_2 | 20.2 ± 1.8 | 20.8 ± 1.5 | 0.152 |
| SMI_1 | 33.8 ± 2.7 | 34.4 ± 2.7 | 0.464 |
| SMI_2 | 33.8 ± 2.7 | 35.3 ± 3.0 | 0.040* |
| TP_1 | 68.8 ± 6.3 | 68.9 ± 5.2 | 0.754 |
| TP_2 | 70.0 ± 6.6 | 68.1 ± 4.1 | 0.287 |
| ALB_1 | 38.8 ± 3.7 | 38.9 ± 4.7 | 0.754 |
| ALB_2 | 39.9 ± 4.7 | 38.0 ± 5.2 | 0.223 |
| PA_1 | 188.6 ± 46.5 | 190.7 ± 40.7 | 0.826 |
| PA_2 | 193.8 ± 47.6 | 188.0 ± 36.1 | 0.884 |
| TRF_1 | 2.6 ± 0.6 | 2.6 ± 0.8 | 0.905 |
| TRF_2 | 2.5 ± 0.6 | 2.4 ± 0.7 | 0.281 |

*1. Mean* ± *SD; 2. Wilcoxon rank sum test*
