# Supplementary material for: Metabolic syndrome worsens sarcopenia and reduces nutritional therapy benefits in advanced gastric cancer
Source: Front Nutr. 2025 Oct 15;12:1615376. doi: 10.3389/fnut.2025.1615376 (PMC12568025; doi:10.3389/fnut.2025.1615376)
Supplement: Supplementary file 1 [file Data_Sheet_1.ZIP › supplementary tables 2025.8.24/supplementary table1_revised 2025.8.19.docx]

**supplementary table1.** Mendelian Randomization Analysis: Assessing Causal Effects in General Populations

| **Variable** | **Source Database** | **GWAS ID** | **No. of SNPs** | **Ancestry** | **Year** | **Website** |
| --- | --- | --- | --- | --- | --- | --- |
| **Exposure (MetS Components)** | | | | | |  |
| Metabolic Syndrome (MetS) | CTGLAB | N/A | 461,902 | European | 2022 | https://cncr.nl/ctg/ |
| Waist Circumference | IEU OpenGWAS | ieu-a-61 | 232,101 | European | 2015 | https://gwas.mrcieu.ac.uk/ |
| Fasting Blood Glucose | IEU OpenGWAS | ebi-a-GCST005186 | 58,074 | European | 2012 | https://gwas.mrcieu.ac.uk/ |
| Hypertension | FinnGen / IEU | finn-b-I9_HYPTENS | 218,754 | European | 2021 | https://gwas.mrcieu.ac.uk/ |
| Triglycerides | IEU OpenGWAS | ieu-a-302 | 177,861 | Mixed | 2013 | https://gwas.mrcieu.ac.uk/ |
| HDL Cholesterol | IEU OpenGWAS | ieu-a-299 | 187,167 | Mixed | 2013 | https://gwas.mrcieu.ac.uk/ |
| **Outcome (Sarcopenia Traits)** | | | | | | |
| Appendicular Lean Mass | UK Biobank | ebi-a-GCST90000025 | 450,243 | European | 2020 | https://gwas.mrcieu.ac.uk/ |
| Hand Grip Strength (Left) | UK Biobank | ukb-b-7478 | 461,089 | European | 2018 | https://gwas.mrcieu.ac.uk/ |
| Hand Grip Strength (Right) | UK Biobank | ukb-b-10215 | 461,089 | European | 2018 | https://gwas.mrcieu.ac.uk/ |
| Usual Walking Pace | UK Biobank | ukb-b-4711 | 459,915 | European | 2018 | https://gwas.mrcieu.ac.uk/ |
